# Antagonistic activity of two *Bacillus* strains against *Fusarium oxysporum* f. sp. *capsici* (*FOC*-1) causing Fusarium wilt and growth promotion activity of chili plant

**DOI:** 10.3389/fmicb.2024.1388439

**Published:** 2024-05-27

**Authors:** Owais Iqbal, Rehana Naz Syed, Nasir Ahmed Rajput, Yi Wang, Abdul Mubeen Lodhi, Rizwan Khan, Sauban Musa Jibril, Muhammad Atiq, Chengyun Li

**Affiliations:** ^1^State Key Laboratory for Conservation and Utilization of Bio-Resources in Yunnan, Yunnan Agricultural University, Kunming, Yunnan, China; ^2^Yunnan-CABI Joint Laboratory for Integrated Prevention and Control of Transboundary Pests, Yunnan Agricultural University, Kunming, Yunnan, China; ^3^Department of Plant Protection, Faculty of Crop Protection, Sindh Agriculture University, Tando Jam, Pakistan; ^4^Department of Plant Pathology, University of Agriculture, Faisalabad, Faisalabad, Pakistan

**Keywords:** chili, Fusarium wilt, *Fusarium oxysporum FOC*-1, *Bacillus*, antagonistic activity, growth promotion activity

## Abstract

*Fusarium oxysporum* f. sp. *capsici* (Foc) poses a significant position in agriculture that has a negative impact on chili plant in terms of growth, fruit quality, and yield. Biological control is one of the promising strategies to control this pathogen in crops. Chili is considered as one of the most important crops in the Hyderabad region that is affected by Fusarium wilt disease. The pathogen was isolated from the infected samples in the region and was confirmed by morphological characteristics and PCR with a band of 488 bp. The bacterial strains were isolated from the rhizosphere soil of healthy plant and also confirmed by PCR with a band of 1,542 bp.The molecular characterization of the fungal and bacterial strain has shown 99.9% homology with the retrieved sequences of *Fusarium oxysporum* f. sp. *capsici* and *Bacillus subtilis* from NCBI. The 1-month-old Ghotki chili plants were inoculated with 1×10^5^ cfu spore/ml^−1^ suspension and confirmed that the *FOC*-1 is responsible for chili Fusarium wilt disease. Subsequently, among the 33 screened *Bacillus* strains, only 11 showed antagonistic activity against *F. oxysporum*. Out of these, only two strains (AM13 and AM21) have shown maximum antagonistic activity against the pathogen by reducing the infection and promoting growth parameters of chili plants under both *in vitro* and greenhouse conditions. The study suggested that biological control is the most promising control strategy for the management of Fusarium wilt of chili in the field.

## 1 Introduction

Chili (*Capsicum annum* L), belongs to Solanaceae family, is one of the most significant crops (Magaña-López et al., 2022) and is widely grown for its spicy taste, pungency, and color. It is a highly rich source of vitamins A and B and is also used in different types of foods, medicines, and cosmetics (Jamil et al., 2021; Akash et al., 2022). In Pakistan, chili crops cover approximately 91,800 hectares of land with an annual production of 115,000 tons (Anjum et al., 2020). The limited yield production of this crop is the main challenge in Pakistan and worldwide. It is very sensitive to various soil borne diseases such as, Fusarium wilt, damping-off and root rot caused by various genera of species, including *Pythium*, *Phytophthora*, *Fusarium*, *Sclerotium*, and *Rhizoctonia* (Dar et al., 2015; Hyder et al., 2021). Among them, Fusarium wilt of chili caused by *Fusarium oxysporum* f. sp. *capsici* is one of the aggressive and damaging diseases, causing huge losses in the crop annually (Velarde-Félix et al., 2018; Tilahun et al., 2024). The pathogen survives for many years in soil debris and has the ability to cause infection from the stage of seedling to fruiting (Shen et al., 2013). After infection, plants showing various symptoms such as dropping leaves, yellowing color, curling, stunted growth, and shorter distances between internodes become dry and eventually lead to death (Shaheen et al., 2021). This pathogen commonly infects the solanaceous crop, especially chili, with 17–22% disease incidence, leading to a decrease in yield by 90.5 to 115.5 thousand tons in Pakistan (Bashir et al., 2018; Serrano-Jamaica et al., 2021).

To combat plant diseases, chemical pesticides and fungicides offer suitable control in the field, but the high use of these chemicals has been reported to become a reason for environmental pollution and may lead to human health issues, such as cancer (Chen and Ying, 2015). The growing interest in this emerging field can be attributed to a widespread desire to decrease dependence on agrochemicals, owing to their adverse impacts on human health and the environment (Iqbal et al., 2023a). Therefore, biological control could be a useful and effective approach to manage wilt disease in both greenhouse and field and will also promote the production of chili crops. Biological control is an alternative method that promotes sustainable and environmentally friendly agricultural practices (Iqbal et al., 2023b). *Trichoderma*, *Paecilomyces*, *Bacillus*, and *Pseudomonas* are most four acceptable genera, comprising more than 200 species which have exhibited remarkable abilities to control a large number of plant diseases while promoting plant growth (Peix et al., 2009; Rivas et al., 2009; Cai et al., 2022; Xu et al., 2023). Out of these, *Bacillus* is one of the most promising biocontrol genus in the agriculture field, controlling soil-borne diseases in many crops (Yuan et al., 2012; Sun et al., 2023), which is also confirmed as plant growth-promoting bacteria (PGPB). *B. subtilis* have the ability to reduce disease incidence and increase plant growth and survival through two different mechanisms such as direct and indirect (Khan et al., 2018). After the successfully suppressed mycelial growth of two soil-borne pathogens in tomato crops, *B. subtilis* PTS-394 was evaluated against the root rot pathogen of chili *Fusarium solani* and found excellent results (Qiao et al., 2023). *B. subtilis* showed broad-spectrum activity against *F. oxysporum*, leading reduced incidence and increased plant growth and parameters in chili crop by producing various antibiotics (Yu et al., 2011). These findings suggest that *B. subtilis* could provide excellent control against chili wilt disease caused by *Fusarium oxysporum* f. sp. *capsici*.

Chili plants were severely infected to wilt disease in Hyderabad region of Sindh province. The infected plants were collected to isolate and identify the causal pathogen responsible for this disease. Through molecular characterization, we confirmed that the causal pathogen is *Fusarium oxysporum* f. sp. *capsici.* We evaluate *Bacillus subtilis* strains against this pathogen under laboratory and greenhouse conditions. The greenhouse experiment was conducted to check the growth promotion and biological activity of chili plants.

## 2 Materials and methods

### 2.1 Sample collection and isolation of the pathogen

During the field survey, chili plants were severely affected with wilt disease and showed symptoms of complete and partial dead plants in Hyderabad region of Sindh province, Pakistan. The infected plants showed stunted growth, minimum fruits, and yellowing to brownish color on the infected leaves. The disease samples were collected in the paper envelop and brought to the Disease Diagnostic Laboratory at the Department of Plant Protection in Sindh Agriculture University, Tando Jam,Hyderabad, Pakistan. The roots of the infected plants were washed with tap water three times. After that, the infected roots were surface sterilized with 70% ethanol and soaked on the filter paper. The roots were cut into small pieces and placed on potato dextrose agar (PDA) containing Petri plates. In total, 1 ml of streptomycin and penicillin antibiotic were mixed in the PDA medium for bacterial contamination. In each Petri plate, five pieces were placed and sealed with parafilm tap and were incubated at 27°C ± 2^0^C for 3 days.

### 2.2 Purification and morphological characteristics of pathogen

The fluffy white, creamy white, or yellowish creamy color colonies were picked carefully and placed on PDA containing plates at the same aforementioned concentration of antibiotics. After ful growth of pathogen, the single hyphae were taken carefully and placed again on the PDA medium plates for purification. The taxonomy of the pathogen was studied carefully with the previous reported literature (Booth, 1971). The picture of microstructures of mycelium, chlamydospore, microconidia and macroconidia were taken under microscope (Carl Zeiss Microimaging GmbH 37,081 Göttingen, GERMANY), to preliminarily clarify their morphological or structural characteristics.

### 2.3 Collection of rhizosphere soil for bacterial strains

To isolate bacterial strains, the rhizosphere soil was collected from five healthy chili plants. The plants were selected randomly and top soil was removed, and then, 15 soil samples were taken from 5–7 inch depth in the plastic bags with the help of soil auger. Next, samples were brought to the Disease Diagnostic Laboratory of the Department of Plant Protection in Sindh Agriculture University, Tandojam, Hyderabad, Pakistan, for the isolation of bacterial strains.

### 2.4 Isolation and purification of bacterial strains from soil

Overall, 5 g of soil sample was taken from each soil sample and soaked for half an hour. After that, the sample was added to 100 ml conical flask containing 45 ml distilled sterilized water and 0.45 g NaCL. The sample was properly shaken in the hot water bath at 37^0^C with 200 rpm for 30 min. After completely mixed, samples were divided into five 2-ml sterilized tubes and were head-shock in the hot water bath at 80^0^C for 10 min. In total, 0.1 ml of suspension from each tube was taken and spread on 90 mm Petri plates containing nutrient agar (NA) medium. The plates were sealed with parafilm tape and were placed upside-down position in the incubator at 37^0^C + 2^0^C for 8–12 h. A milky white, creamy white, and yellowish white streaky color colonies were visually examined with different size or shapes on the plates. Colonies were picked carefully to re-streak on NA medium for purification. All strains were preserved at −20°C for further study. To identify the *Bacillus* strains, their morphology was evaluated based on colony type, bacterial shape, size, and growth characteristics in the NA medium (Bergey, 1994).

### 2.5 Screening of bacterial strains

A total of 33 bacterial strains were screened by the dual assay method for their antagonistic activity on PDA medium against *Fusarium oxysporum* f. sp. *capsici FOC*-1 (Gupta et al., 2001). A filter paper was cut in 5 mm size and 3 pieces were placed on PDA plate at 90^0^ angle. Two strains were tested in each plate. In total, 0.3 μl of actively grown bacterial suspension was swamped on two filter papers and 0.3 μl of distilled sterilized water (ddH2O) was flooded on one filter paper for control (Utkhede and Sholberg, 1986). The plates were sealed with parafilm tape and incubated at 28^0^C + 2^0^C for 2 days as overturned position. The plates were examined on a daily basis, and growth inhibition zone (GIZ) of fungus was determined in diameter.

### 2.6 Antagonistic activity

Among 33 bacterial strains, 11 bacterial strains were selected based on screening, and their antagonistic activity was checked against *FOC*-1 as the aforementioned method of these 11 strains. All bacterial strains were grown on NA medium as aforementioned method. Next, the strains were used against the pathogen on PDA medium. The growth inhibition zone (GIZ) was recorded as the aforementioned method. Out of these 11 strains, 2 strains that have shown maximum biological potential activity against the pathogen were selected for the greenhouse study of chili plant growth promotion.

### 2.7 Pathogenicity assay

The Ghotki Chili variety seeds were grown in the incubator at room temperature (30^0^C + 2^0^C) and transferred in the thermopole pots of 8 cm in size. Next, a 30 mm water added in the purified plates of pathogen and scratched mycelia with the help of sterile applicator for fungal suspension. The hemocytometer was used to adjust to 1 × 10^5^ spore per ml colony forming unit (CFU) concentration of pathogen suspension. The 1-month-old chili plants were inoculated with 1 × 10^5^ cfu spore per ml suspension. The inoculated plants were observed continuously. After 7–10 days of inoculation, symptoms of the disease were observed on chili plants. The infected plants were taken to the laboratory, and then photograph of the diseased plants was taken; causal pathogen was successfully re-isolated from infected plant roots using the aforementioned method. Finally, the pathogenicity was confirmed based on Koch’s postulate.

### 2.8 Molecular identification

#### 2.8.1 DNA extraction

For the extraction of DNA, a CTAB method developed by Doyle and Doyle (1987) was utilized with slight modification from the biocontrol agent culture and other microorganisms. A nanodrop spectrophotometer was used to check the DNA concentration and purity using method by Li et al. (2006). Furthermore, the DNA concentration and purity were analyzed by running the samples on 1% agarose gel for 30 min.

#### 2.8.2 PCR-based detection

In PCR-based detection, a pair of primers, ITS1 and ITS4, was evaluated to amplify the pathogen, and 16 s RNA sequence primer was used for bacteria (White et al., 1990). The PCR reactions including reagents were 1.5 μl of each primer, 7 μl of master mix, and 0.5 μl of Platinum Taq-polymerase in a total volume of 12.5 μl of reaction. An automated thermal cycler was employed to conduct the PCR amplification with a protocol consisting of an initial denaturation at 96°C for 9 min, followed by 40 cycles of denaturation at 96°C for 30 s and annealing at 53°C for 1 min. The final extension was carried out at 72°C for 7 min. The amplified products were detected on a 1.5% agarose gel containing ethidium bromide (Li et al., 2006).

#### 2.8.3 Characterization of the strains

The manufacturer’s recommendations (Bio Product) were followed in sequencing the PCR-amplified products that were positive. BioEdit version 7.2 software was used to analyze the sequences (Hall, 1999) and compared with the retrieved sequences from (NCBI) blast tool. After that, the sequence was uploaded to MEGA-7 software and align with the help of ClustalW program (Kumar et al., 2016). A phylogenetic tree was constructed with the help of neighbor joining method with 1,000 bootstrap value and Tamura 3-parameter model (Kong et al., 2000).

### 2.9 Growth promotion and biological activity

In this experiment, two bacterial strains were selected on the basis of high antagonistic activity against *Fusarium oxysporum* f. sp. *capsici FOC*-1. The Ghotki chili variety seeds were collected from a nearby shop and brought to the laboratory. The seeds were washed three times with distilled water and surface sterilized with 70% ethanol. Next, 20 seeds were dipped in the bacterial suspension for growth promotion and biological assay for 30 min and dried on sterilized blotting paper. Similarly, the control seeds were dipped in sterilized distilled water (ddH2O) for 30 min. The seeds were placed on sterilized filter paper in 90 mm petri plate and incubated at (30^0^C + 2^0^C) for 3 days. After growing in the incubator, the five seedlings were transferred to each 80 cm soil pot containing sterilized soil with peat moss at a ratio of 3:1. The plants were examined regularly, and 50 ml ddH2O water was added on a daily basis. In the biological assay, 1×10^5^ cfu spore/ml of pathogen suspension was placed in the roots of plants after 15 days. After 1 month, data of plant parameters were recorded, such as root length, shoot length, fresh weight, and dry weight.

### 2.10 Data analysis

Statistical parameters such as mean, standard deviation, analysis of variance and LSD multiple comparison tests were calculated using the Statisix-8.1 package. The GraphPad prism 8 version was used to develop the graph and edited or merged with the help of Adobe Illustrator CC 2019.

## 3 Results

### 3.1 Isolation and purification of pathogen

During the field visit investigation, chili plants severely infected by wilt disease and has shown symptoms of complete and partial dead plants in Hyderabad region of Sindh province, Pakistan. The infected chili plants showed yellowish and brownish colors on the leaves as compared with healthy plants. Infected plants also showed stunted growth having small number of fruits. On PDA medium, the infected roots have shown a number of creamy to whitish creamy colonies. These isolates showed similar morphological characteristics to *Fusarium oxysporum* f. sp. *capsici* and were given the isolate name of *FOC-*1.

### 3.2 Microscopic and morphological study

**Figure 1:**
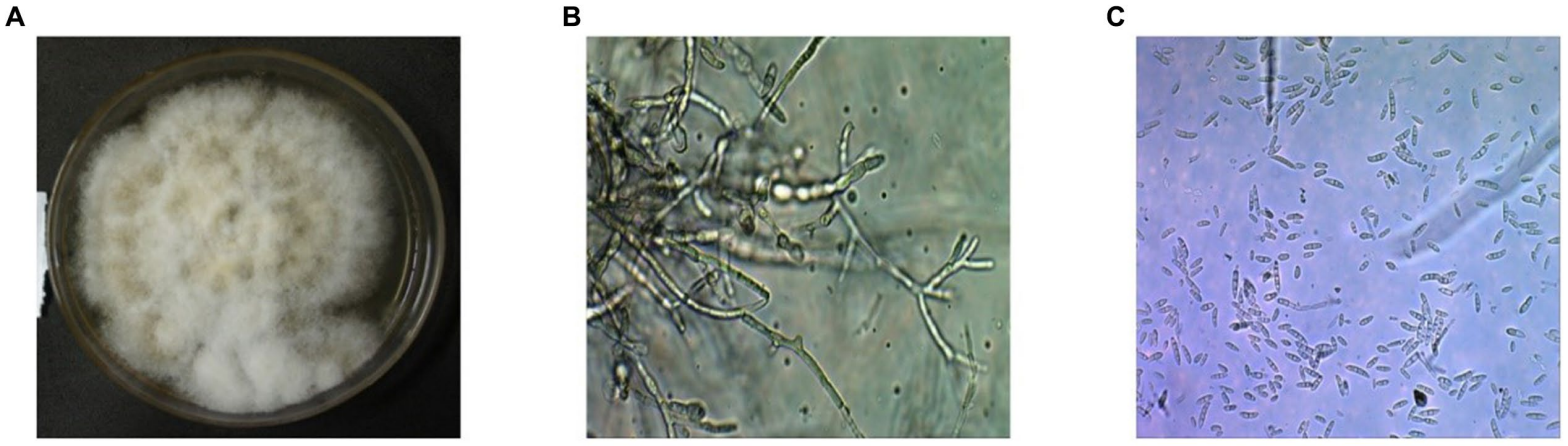
Microscopic and macroscopic structures on the PDA plate of the isolated fungus *Fusarium oxysporum* f. sp. *capsici FOC-*1 from Hyderabad, Sindh, Pakistan. **(A)** Mycelial growth of the *FOC*-1 on PDA medium **(B)** conidiophores and **(C)** microconidia and macroconidia of the pathogen under microscope. The 3.0 USB camera microscope was used for chlamydospore, microconidia and macroconidia pictures.

After 5 days of incubation period, fluffy whitish to yellowish creamy colonies were grown with septate mycelium and hyaline frequently branched on the PDA plates. Conidia are asexual spores produced by the fungus. The fungus produced microconidia and macroconidia on the PDA plates with different size and shape, but they all are colorless. The conidiophores displayed a range of sizes and shapes, including both simple and stout and slender structures (Figures 1A–C). Microconidia of the isolates typically single celled and slightly curved with size of 5–12 × 2.3–3.5 μm. However, the macroconidia are long with 3–5 septate, bent, and slightly curved at the end of pointed with size of 27–46 × 3–4.5 μm, respectively.

### 3.3 *In vitro* efficacy of bacterial strains

During the present study, first, we screened all 33 bacterial strains for their potential activity against *Fusarium oxysporum* f. sp. *capsici FOC*-1 on a potato dextrose agar (PDA) medium using the dual culture technique (Nakkeeran et al., 2006). Out of 33 strains, only 11 strains (AM03, AM05, AM08, AM11, AM13, AM17, AM19, AM21, AM23, AM26, and AM29) have shown antagonistic activity, while the other 22 strains did not show any antifungal activity against the tested pathogen (Table 1).

**Table 1:** *In vitro* screening of 33 bacterial strains against *Fusarium oxysporum* f. sp. *capsici FOC*-1 using the plate culture method on PDA medium.

**No#**	**Isolates**	**Antagonistic activity against *Fusarium oxysporum* f. sp. *capsici FOC-*1**
01	AM01	---
02	AM02	---
03	AM03	+
04	AM04	---
05	AM05	++
06	AM06	---
07	AM07	---
08	AM08	++
09	AM09	---
10	AM10	---
11	AM11	+
12	AM12	---
13	AM13	+++
14	AM14	---
15	AM15	---
16	AM16	---
17	AM17	+
18	AM18	---
19	AM19	++
20	AM20	---
21	AM21	++++
22	AM22	---
23	AM23	++
24	AM24	---
25	AM25	---
26	AM26	++
27	AM27	---
28	AM28	---
29	AM29	++
30	AM30	---
31	AM31	---
32	AM32	---
33	AM33	---

The width of growth inhibition zone GIZ is as follows. Three --- represents 0 mm growth inhibition, one + represents 1–10 mm, two ++ represents 11–20 mm, three +++ represents 21–30 mm and four ++++ represents 31–40 mm growth inhibition.

### 3.4 Antagonistic activity of bacterial agents

**Figure 2:**
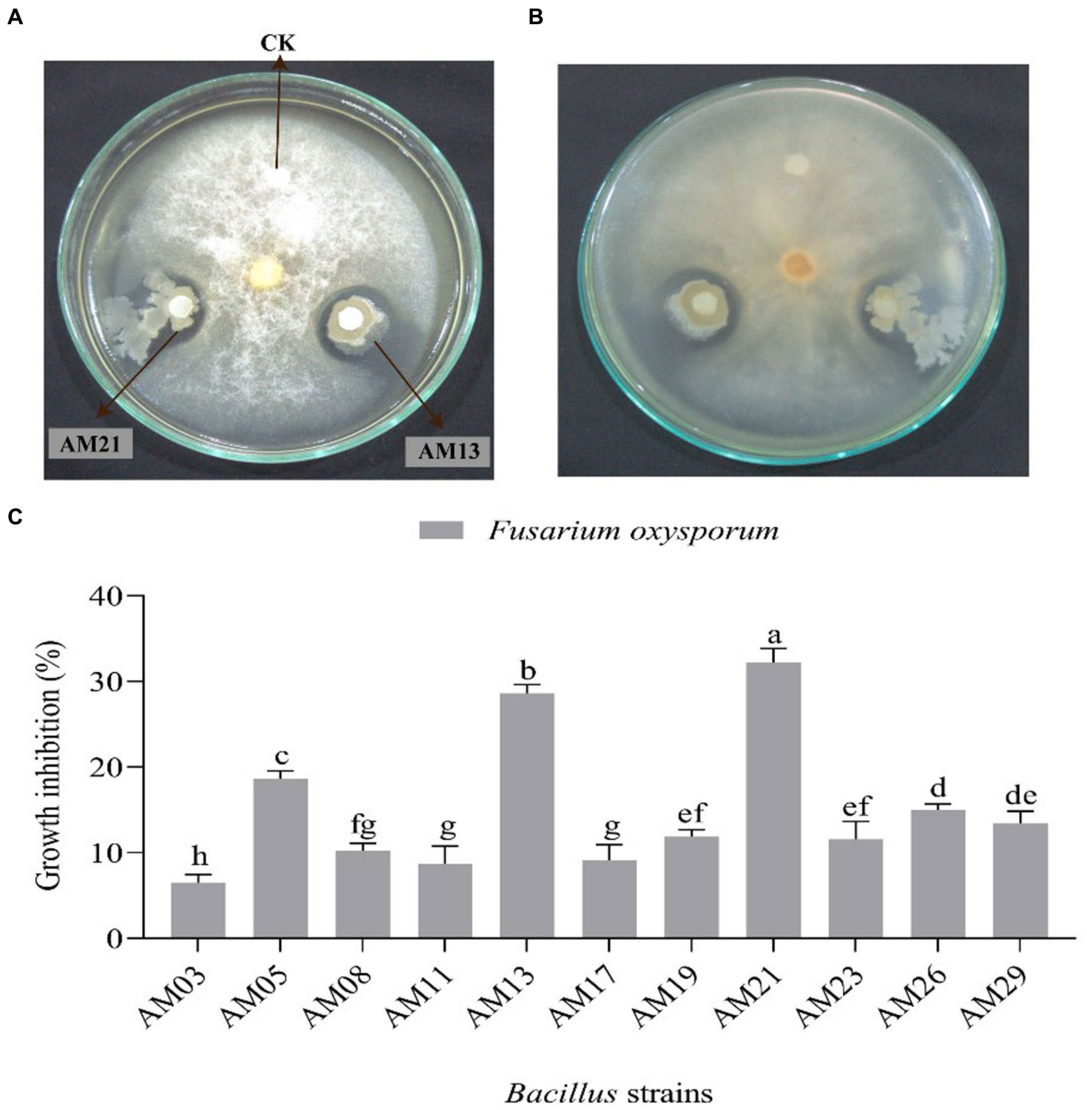
Antagonistic activity of two different *Bacillus* strains against *Fusarium oxysporum FOC*-1 under laboratory condition. **(A)** Show the front image of the dual assay plate method **(B)** show the opposite plate direction **(C)** growth inhibition percentage of 11 bacterial strains against *F. oxysporum*. The error bars and different letters represent the least significant difference value at p = 0.05.

Among the 11 tested bacterial strains, 2 *Bacillus* strains, namely, AM13 and AM21 were found excellent against *FOC*-1. Both strains show highly antagonistic activity against the tested pathogen and successfully inhibited the mycelial growth under plate condition (Figures 2A,B). The highest (32.21%) growth inhibition zone (GIZ) was recorded with AM21 followed by AM13 (28.61%). However, the other bacterial strains showed moderately and least effective antagonistic activity against the tested pathogen. The minimum (6.51%) GIZs was recorded with AM03 followed by AM11 (8.72%), AM17 (9.11%), AM08 (10.24%), AM23 (11.57%), AM21 (11.88%), AM26 (13.43%), AM19 (15.03%), and AM05 (18.66%), respectively (Figure 2C).

### 3.5 Pathogenicity test

**Figure 3:**
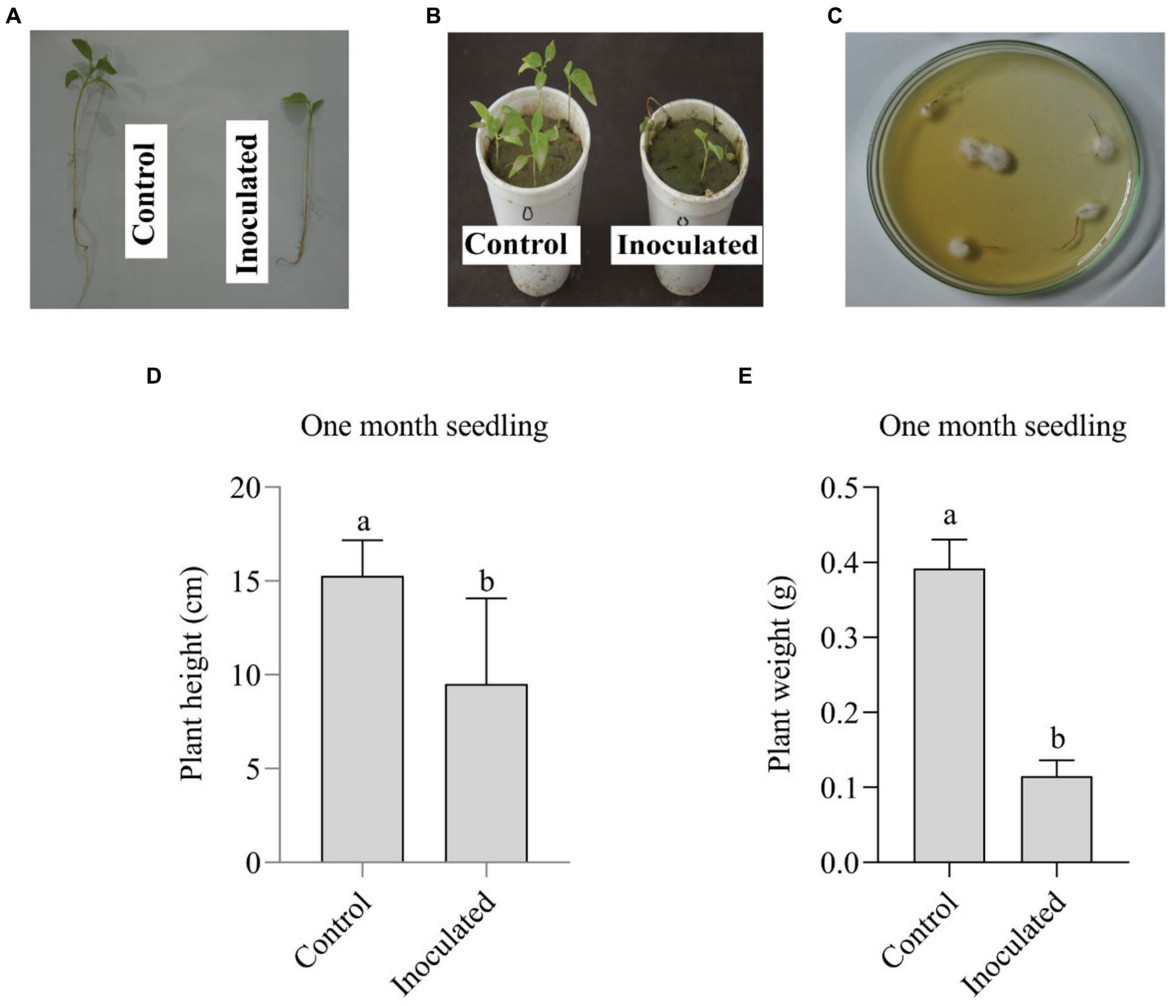
Pathogenicity assay on the 1-month-old chili seedling plants to check the effect of *FOC*-1. **(A,B)** Un-inoculated control plant was observed healthy and the inoculated plant showed stunted growth, wilting symptoms, and **(C)** completely recovered *FOC*-1 isolate from infected roots of inoculated plants. **(D,E)** Showed plant height and weight of the control and inoculated plants. Letter and bars significantly differ (*p* < 0.05).

In the pathogenicity assay, 1-month-old chili plants were inoculated with *FOC*-1 conidial suspension (cfu 1×10^5^ spore/ml). The 1-month-old chili plants infected by the *FOC*-1 have produced various symptoms on plants by causing partially and completed mortality as compared with un-inoculated control plants, while inoculated plants show minimum plant height, weight, stunted growth, and yellowing to brownish color wilting symptoms on the leaves (Figures 3A,B). For confirmation of *FOC*-1 infection, the isolate was successfully recovered on PDA medium with high frequency (Figure 3C). In addition, the highest plant height (15.270 cm) was recorded with control followed by *FOC*-1-inoculated plants (9.5 cm). Similarly, lowest plant weight (0.115 g) was recorded with *FOC*-1 plant followed by control plants (0.392 g), respectively (Figures 3D,E).

### 3.6 Molecular characterization

**Figure 4:**
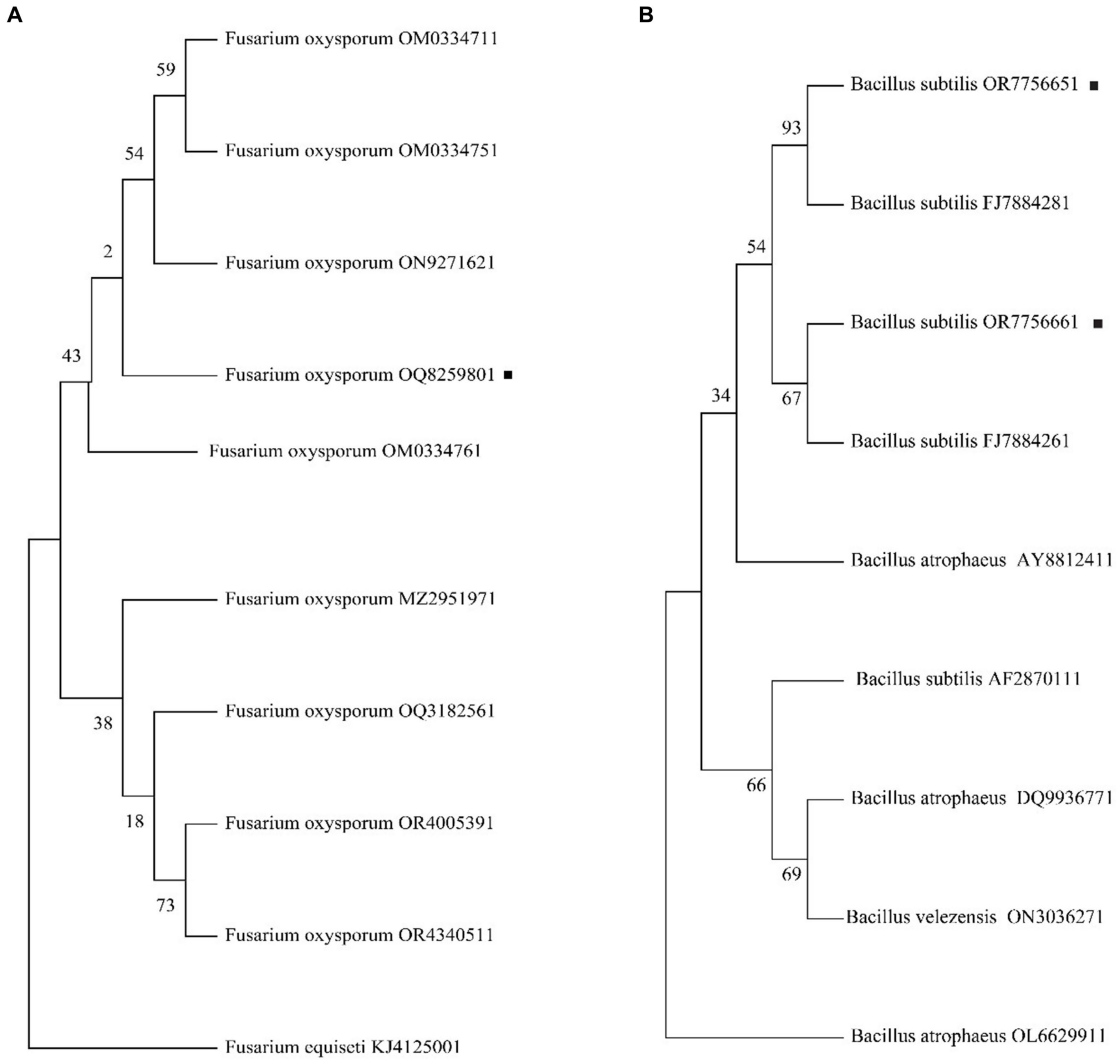
Phylogenetic analysis of *FOC*-1 isolates from infected chili plants. **(A)** *Fusarium oxysporum* f. sp. *capsici* FOC-1 tree **(B)** *Bacillus subtilis* AM13 and AM21 tree. The maximum likelihood program in MEGA 11 software was used for phylogenetic tree with partial 488 bp and 1,542 bp sequences. The black dot represents the *F. oxysporum FOC*-1 ITS and *B. subtilis* AM13 and AM21 16 s sequences.

The internal transcribed spacer (ITS) amplification products for *FOC*-1 showed that all fragments were 488 bp in length (Supplementary Figure S1), and the 16 s RNA amplified products of AM13 and AM21 were 1,500 bp in length. The obtained PCR product was sequenced and compared with NCBI BLAST sequences, which has shown 99.9% sequence similarity to the GenBank *Fusarium oxysporum* f. sp. *capsici* (OM033476) and *Bacillus subtilis* (FJ788428 and FJ788426) sequence as the polygenetic tree shown in Figures 4A,B. The molecular study results confirmed that the *FOC*-1 isolate was very similar to *F. oxysporum* f. sp. *capsici,* causal agent of chili wilt disease, and AM13 and AM21 were most similar to *B. subtilis*. The sequences submitted in the NCBI GenBank with (OQ825980, OR775665, and OR775666) association numbers.

### 3.7 Plant growth promotion under greenhouse

**Figure 5:**
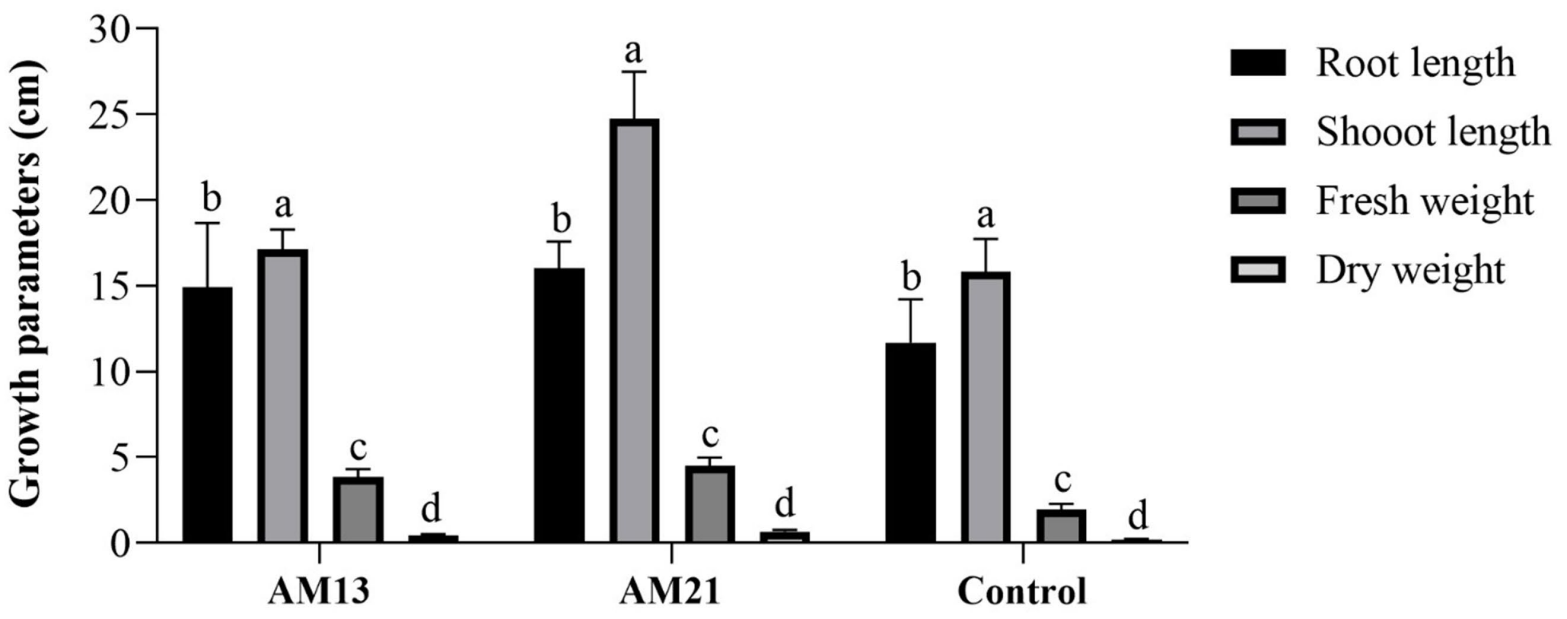
Growth promotion activity of two *Bacillus* strains in chili plants. Letter and bars showed standard deviation and significantly differ (*p* < 0.05).

Two *Bacillus* strains which had shown maximum antagonistic activity against *FOC*-1 *in vitro* were used for growth promotion and biological activity of chili plant. Both strains proved highly effective and enhanced plant parameters as compared to control. The maximum root length (16 cm), shoot length (24.74 cm), fresh weight (4.51 g), and dry weight (0.662 g) were recorded in treated plants with AM21 followed by AM13 with root length (14.93 cm), shoot length (17.12 cm), fresh weight (3.84 g), and dry weight (0.46 g), respectively. The minimum growth parameters such as root length (11.7 cm), shoot length (15.83 cm), fresh weight (1.93 g), and dry weight (0.196 g) were recorded in the control plants (Figure 5).

### 3.8 Biological activity

Bacterial suspension-coated seeds of Ghotki chili variety were grown in the incubator and transferred to the 8 cm thermopole pots in greenhouse. The pathogen *FOC*-1 suspension was applied to the roots after 15 days. In comparison to control, both bacterial strains AM13, and AM21, enhance the growth of plants and show excellent biological control against *FOC*-1, respectively (Table 2).

**Table 2:** Biological activity of AM-13 and AM-21 in 1-month-old chili plants against *Fusarium oxysporum* f. sp. *capsici FOC*-1.

Treatments	Root length	Shoot length	Fresh weight	Dry weight
AM13 + *FOC*-1	12.33 ± 1.064b	16 ± 1.9b	1.696 ± 0.355b	0.221 ± 0.038b
AM21 + *FOC*-1	13.36 ± 0.305a	19.7 ± 0.264a	2.573 ± 0.236a	0.380 ± 0.021a
*FOC*-1	7.96 ± 2.291d	10.66 ± 1.113d	0.930 ± 0.380d	0.049 ± 0.015d
Control	10.93 ± 0.360c	13.72 ± 0.818c	1.590 ± 0.736c	0.151 ± 0.023c

The letters and values significantly differ (*p*-value < 0.05). a* presented a highly effective. b* presented an effective. c* presented a moderately effective. d* presented a least effective.

## 4 Discussion

Fungal pathogens represent a substantial menace to agriculture, crop yield, and global food production (Singh et al., 2023). The genus *Fusarium* is known to cause wilt disease of over 100 plant species and is ranked fifth deadliest plant pathogen (Dongzhen et al., 2020; Rampersad, 2020; Medeiros-Araujo et al., 2021; Girma, 2022). Fusarium wilt of chili ranks as the third most devastating disease affecting chili crops. Currently, agrochemical products are the predominant methods employed for disease control. Nevertheless, the excessive application of these chemicals not only poses adverse effects on the environment and human health but also targets beneficial life forms in the field (Ramesh et al., 2009; Tudi et al., 2021). Biological control methods have emerged as effective and environmentally friendly alternatives that have garnered significant attention and are rapidly being adopted to replace chemical control measures (Narasimhan and Shivakumar, 2015; Baker et al., 2020). Biocontrol agents offer the advantage of easy transfer to the field and have the potential to augment host resistance, immunity, plant growth, yield, and biomass production. Among these agents, *Bacillus* species stand out as widely utilized against various pathogens, renowned for their ability to enhance plant growth, induce resistance through the production of antimicrobial compounds, and generate secondary metabolites (Singh et al., 2017; Miljaković et al., 2020; Bamisile et al., 2021). In the current investigation, a survey was conducted in chili fields in the Hyderabad region of Pakistan to identify the causative agent of the disease. Using isolation techniques, the *FOC*-1 isolate was successfully identified from infected plant roots. Employing Koch’s postulates, *FOC*-1 was confirmed as the pathogenic fungus which was responsible for chili wilt disease in this region. Subsequent morphological and molecular characterization validated the pathogen *F. oxysporum* f. sp. *capsici*, which causes wilt disease in Solanaceae crops, as described in the literature (Menge et al., 2020; El-Kazzaz et al., 2022). Current findings unveiled the intricate interplay between *Bacillus* strains and pathogenic fungus in the context of Fusarium wilt management and chili plant growth promotion. Screening of 33 bacterial strains for antagonistic activity against *FOC*-1 on PDA medium identified 11 strains with significant inhibition of mycelial growth. *Bacillus* strains AM13 and AM21 exhibited the highest antagonistic activity, with growth inhibition zones (GIZs) of 28.61 and 32.21%, respectively, while other strains showed varying degrees of effectiveness. In pathogenicity tests, *FOC*-1 inoculation of 1-month-old chili plants resulted in symptoms including reduced plant height, weight, stunted growth, and wilting of leaves. The highest plant height (15.270 cm) was recorded in control plants, while *FOC*-1-inoculated plants exhibited the lowest height (9.5 cm). Similarly, the lowest plant weight (0.115 g) was recorded in *FOC*-1-inoculated plants, contrasting with the highest weight (0.392 g) in control plants. Under greenhouse conditions, treatment with AM21 resulted in the maximum root length (16 cm), shoot length (24.74 cm), fresh weight (4.51 g), and dry weight (0.662 g) in treated plants, followed by AM13 with root length (14.93 cm), shoot length (17.12 cm), fresh weight (3.84 g), and dry weight (0.46 g). Control plants exhibited the minimum growth parameters, with root length (11.7 cm), shoot length (15.83 cm), fresh weight (1.93 g), and dry weight (0.196 g). Biological activity assays demonstrated the efficacy of *Bacillus* strains AM13 and AM21 in enhancing plant growth and providing biological control against *FOC*-1, as evidenced by improved growth parameters compared with controls. The present study is supported by previous studies that demonstrated the quantitative effectiveness of bacterial antagonists in inhibiting the radial growth of phytopathogens. The previous research efforts showed an antagonistic efficacy of 56.2% against *Fusarium oxysporum* and 51.02% against *Alternaria alternata*, which aligns with our findings of *Bacillus* strains, effectively inhibiting the mycelial growth of *FOC*-1 (Chandra et al., 2020; Kumar et al., 2020). *In vitro* antagonism trial depicted that approximately 88% endophytic isolates minimized the mycelial growth of *F. oxysporum* (41%) as compared with *R. solani* (24%) and *P. aphanidermatum* (30%). The application of bacterial endophytes reduced disease incidence by 70% and improved the fresh biomass of roots (2.33-fold) and shoots (3.80-fold) compared with pathogen control plants. These numerical values support the effectiveness of *Bacillus* strains in enhancing plant growth parameters and combating *F. oxysporum* infection, consistent with our current findings (Gupta et al., 2022). In a previous research endeavor, the utilization of *Bacillus subtilis* CAS15 strongly advocated the current findings. *Bacillus subtilis* CAS15 strain exhibited a strong ability to inhibit the mycelial growth of 15 plant fungal pathogens, with rates ranging from 19.26 to 94.07%. Additionally, CAS15 significantly reduced the incidence of Fusarium wilt in pepper plants by 12.5–56.9%, indicating its potential to induce systemic resistance. Moreover, treated plants showed notable increases in height at various stages, ranging from 27.24 to 54.53% taller than controls. Furthermore, CAS15 enhanced pepper yield by shortening the time to 50% flowering to 17.26 days, increasing average fruit weight by 36.92% and boosting average yield per plant by 49.68% (Yu et al., 2011). Another study screened 59 PGPR against *Colletotrichum truncatum,* and only 18 PGPR successfully suppressed mycelial growth of the tested pathogen and 8 PGPR enhanced growth promotion activity of chili plant. Among the PGPR isolates, seed treatment with *Bacillus amyloliquefaciens* resulted in significant enhancements in seed germination (84.75%) and seedling vigor (1423.8), which was accompanied by increased vegetative growth parameters in chili plants. Furthermore, greenhouse experiments demonstrated substantial disease protection, with a remarkable 71% reduction in anthracnose disease incidence observed in plants pretreated with *B. amyloliquefaciens*, followed by *B. cepacia* and *P. rettgeri*. This induced resistance was supported by higher activity levels of defense enzymes (phenylalanine ammonia lyase, peroxidase, polyphenol oxidase, and β-1,3-glucanase) (Gowtham et al., 2018). *B. subtilis* has been reported to produce volatile compounds such as indole acetic acid, siderophores, amylase, extracellular protease, cellulose, and β-1,3-glucanase, enhancing the defense-related enzyme activities for PPO, SOD, CAT, PAL, and LOX and growth promotion in various crops (Shasmita Swain et al., 2022; Xu et al., 2022; Yang et al., 2023). In other previous studied cases, similar findings were reported where only one bacterial strain, *Bacillus subtilis* APK, exhibited significant antifungal potential against the anthracnose pathogen. This *Bacillus* strain demonstrated decreased pathogen mycelial growth *in vitro* and enhanced chili seedling growth under greenhouse conditions (Kumar et al., 2021). Furthermore, a previous research has indicated that several *Bacillus* species can notably improve the growth and development of chili (Peña-Yam et al., 2016). These consistent results underscore the efficacy of *Bacillus* strains in both combating fungal pathogens and promoting the growth of chili plants.

## 5 Conclusion

*Fusarium oxysporum* f. sp. *capsici* is one the most destructive and devastating pathogens of chili crop. The present study evaluated two *Bacillus* strains having antagonistic activity against *F. oxysporum* and that ultimately lead to growth promotion in chili plant. It is recommended that these strains could be used as part of the integrated management system to provide effective control of this disease. It is also suggested to use these strains against other plant pathogens and may provide as safest management control as compared with chemical pesticides.

## Data availability statement

The datasets presented in this study can be found in online repositories. The names of the repository/repositories and accession number(s) can be found at: https://www.ncbi.nlm.nih.gov/, OQ8259801, OR7756651 OR7756661.

## Author contributions

OI: Data curation, Investigation, Methodology, Writing – original draft. RS: Conceptualization, Writing – review & editing. NR: Conceptualization, Writing – review & editing. WY: Writing – review & editing. AL: Data curation, Formal analysis, Writing – review & editing. RK: Formal analysis, Writing – review & editing. SJ: Writing – review & editing. MA: Writing – review & editing. CL: Writing – review & editing.
