# Supplementary material for: Antagonistic activity of two Bacillus strains against Fusarium oxysporum f. sp. capsici (FOC-1) causing Fusarium wilt and growth promotion activity of chili plant
Source: Front Microbiol. 2024 May 27;15:1388439. doi: 10.3389/fmicb.2024.1388439 (PMC11163047; doi:10.3389/fmicb.2024.1388439)
Supplement: Supplementary file 1 [file Table_1.DOCX]

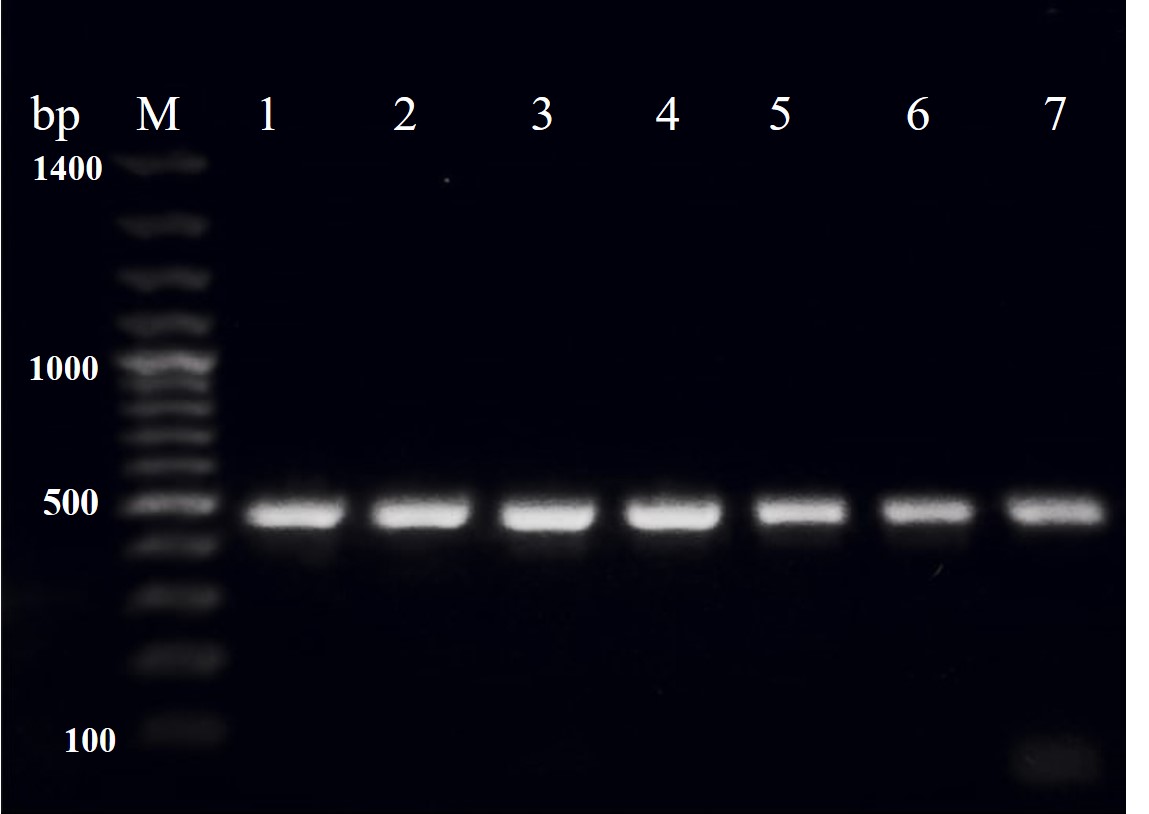


Supplementary Fig. 1. PCR amplification of genomic DNA extracted from FOX-1 revealed that all the resulting fragments had a length of 488 base pairs. In the experiment, a marker (M) was used as a size reference, and the DNA samples from seven FOX-1 samples were loaded in lanes numbered 1 to 7.
